# Draft Genome Sequence of *Staphylococcus hominis* BHG17 Isolated from Wild Bar-Headed Goose (*Anser indicus*) Feces

**DOI:** 10.1128/genomeA.01552-16

**Published:** 2017-02-02

**Authors:** Wen Wang, Si-Si Zheng, Kirill Sharshov, Hao Sun, Xian-Qiang Wu, Fang Yang, Xue-Lian Wang, Lai-Xing Li

**Affiliations:** a Key Laboratory of Adaptation and Evolution of Plateau Biota, Northwest Institute of Plateau Biology, Chinese Academy of Sciences, Xi’ning, China; b Center of Growth, Metabolism and Aging, College of Life Sciences, Sichuan University, Chengdu, China; c University of the Chinese Academy of Sciences, Beijing, China; d Research Institute of Experimental and Clinical Medicine, Novosibirsk, Russia

## Abstract

*Staphylococcus hominis* belongs to a group of coagulase-negative staphylococci and is an opportunistic pathogen, usually found on the skin and mucous membranes. Studies involving *S. hominis* isolated from wild birds are scarce. Here, we report a 2.365-Mb draft genome sequence of *S. hominis* BHG17, isolated from the feces of a bar-headed goose.

## GENOME ANNOUNCEMENT

Coagulase-negative staphylococci (CoNS) are the indigenous microbiota of the human skin and mucous membrane and are currently one of the most important human pathogens, with the potential to cause nosocomial bloodstream infections related to implanted medical devices (1, 2). *Staphylococcus hominis* is the third most commonly isolated species among CoNS from clinical cases (3). *S. hominis* is the major pathogen causing bacteremia, septicemia, endophthalmitis, and endocarditis (4, 5). Furthermore, this organism has been reported to exhibit resistance to the most frequently used antimicrobials such as macrolides, lincosamides, vancomycin, and streptogramins (6, 7). This limits the therapeutic options available and makes an *S. hominis* infection a serious threat to public health. Many strains of *S. hominis* have been isolated from humans, environments, and mosquitoes (8, 9). However, there is a lack of data about the presence of *S. hominis* in wild birds. In this direction, the work here presents the draft genome sequence of *S. hominis* BHG17, which was isolated from the feces of a wild bar-headed goose.

The whole-genome DNA was extracted and then sequenced using the Illumina HiSeq 4000 platform (one lane, 2 × 150-bp paired-end run). A total of 11,359,726 paired-end reads, with lengths of 150 nucleotides, of 1,703,958,900 bases were generated, which, after quality control, resulted in 10,865,870 clean reads of 1,554,269,893 bp. *De novo* assembly of these clean reads was carried out using the newly developed SPAdes version 3.9.0 algorithm (10) followed by error correction using the Bayes Hammer program (11). The reads were assembled into 43 contigs, with an *N*_50_ value of 96,370 bp and a largest contig size of 247,721 bp. Then, gene prediction was performed with Prodigal version 2.6.3 (12), while tRNAs were predicted with tRNAScan-SE version 1.3.1 (13), and rRNAs were predicted with RNAmmer version 1.2 (14). Subsequently, the gene functions were annotated into the KEGG (15) and COG (16) databases using NCBI BLAST version 2.2.31+.

The genome is 2,365,478 bp in length, with a G+C content of 31.43%. This microbe possesses 2,451 genes, 2,326 coding sequences (CDSs), 57 tRNAs, and eight rRNAs. Approximately 76.31% (*n* = 1,775) of the CDSs were assigned to functional clusters of orthologous groups, and 54.17% (*n* = 1,260) were assigned a KEGG orthology number. The genomes of strain BHG17 include three genes associated with specific resistance to lincomycin (*lnuA*), macrolide (*mphC*), and lincosamide-macrolide-streptogramin_b (*msrA*), respectively. Overall, the availability of the present genome sequences facilitates further bioinformatics analysis in *S. hominis* populations.

### Accession number(s).

This whole-genome shotgun project has been deposited in GenBank under the accession number MPNR00000000. The version described in this paper is the first version, MPNR01000000.
